# Supplementary material for: Resolving the full spectrum of human genome variation using Linked-Reads
Source: Genome Res. 2019 Apr;29(4):635–45. doi: 10.1101/gr.234443.118 (PMC6442396; doi:10.1101/gr.234443.118)
Supplement: Supplemental Material [file supp_gr.234443.118_Supplemental_longranger-2.2.zip › longranger-2.2.tar.bz2!/lr2.2-public/lib/stan/cmdstan/stan/doc/api/html/classstan_1_1agrad_1_1chainable.html]

Stan: stan::agrad::chainable Class Reference


|  |  |
| --- | --- |
|  | Stan  2.5.0  probability, sampling & optimization |


- Main Page
- Related Pages
- Namespaces
- Classes
- Files

- Class List
- Class Index
- Class Hierarchy
- Class Members

All Classes Namespaces Files Functions Variables Typedefs Enumerations Enumerator Friends Macros Pages

- stan
- agrad
- chainable

Public Member Functions |
Static Public Member Functions |
List of all members

stan::agrad::chainable Class Reference

Abstract base class for variable implementations that handles memory management and applying the chain rule.
More...

`#include <chainable.hpp>`

Inheritance diagram for stan::agrad::chainable:

|  |  |
| --- | --- |
| Public Member Functions | |
|  | chainable () |
|  | Construct a chainable object. More... |
|  | |
| virtual | ~chainable () |
|  | Chainables are not destructible and should go on the function call stack or be allocated with operator new. More... |
|  | |
| virtual void | chain () |
|  | Apply the chain rule to this variable based on the variables on which it depends. More... |
|  | |
| virtual void | init\_dependent () |
|  | Initialize this chainable's adjoint value to make it the dependent variable in a gradient calculation. More... |
|  | |
| virtual void | set\_zero\_adjoint () |
|  | Set the value of the adjoint for this chainable to its initial value. More... |
|  | |

|  |  |
| --- | --- |
| Static Public Member Functions | |
| static void \* | operator new (size\_t nbytes) |
|  | Allocate memory from the underlying memory pool. More... |
|  | |
| static void | operator delete (void \*) |
|  | Delete a pointer from the underlying memory pool. More... |
|  | |

## Detailed Description

Abstract base class for variable implementations that handles memory management and applying the chain rule.

Definition at line 14 of file chainable.hpp.

## Constructor & Destructor Documentation

|  |  |  |  |  |
| --- | --- | --- | --- | --- |
| stan::agrad::chainable::chainable | ( |  | ) |  |

inline

Construct a chainable object.

The implementation in this abstract base class is a no-op.

Definition at line 22 of file chainable.hpp.

|  |  |  |  |  |
| --- | --- | --- | --- | --- |
| virtual stan::agrad::chainable::~chainable | ( |  | ) |  |

inlinevirtual

Chainables are not destructible and should go on the function call stack or be allocated with operator new.

Definition at line 28 of file chainable.hpp.

## Member Function Documentation

|  |  |  |  |  |
| --- | --- | --- | --- | --- |
| virtual void stan::agrad::chainable::chain | ( |  | ) |  |

inlinevirtual

Apply the chain rule to this variable based on the variables on which it depends.

The base implementation in this class is a no-op.

Reimplemented in stan::agrad::precomputed\_gradients\_vari, stan::agrad::stored\_gradient\_vari, stan::agrad::partials\_vari, and stan::agrad::precomp\_v\_vari.

Definition at line 37 of file chainable.hpp.

|  |  |  |  |  |
| --- | --- | --- | --- | --- |
| virtual void stan::agrad::chainable::init\_dependent | ( |  | ) |  |

inlinevirtual

Initialize this chainable's adjoint value to make it the dependent variable in a gradient calculation.

Reimplemented in stan::agrad::vari.

Definition at line 44 of file chainable.hpp.

|  |  |  |  |  |  |
| --- | --- | --- | --- | --- | --- |
| static void stan::agrad::chainable::operator delete | ( | void \* |  | ) |  |

inlinestatic

Delete a pointer from the underlying memory pool.

This no-op implementation enables a subclass to throw exceptions in its constructor. An exception thrown in the constructor of a subclass will result in an error being raised, which is in turn caught and calls delete().

See the discussion of "plugging the memory leak" in: http://www.parashift.com/c++-faq/memory-pools.html

Definition at line 77 of file chainable.hpp.

|  |  |  |  |  |  |
| --- | --- | --- | --- | --- | --- |
| static void\* stan::agrad::chainable::operator new | ( | size\_t | *nbytes* | ) |  |

inlinestatic

Allocate memory from the underlying memory pool.

This memory is is managed by the gradient program and will be recovered as a whole. Classes should not be allocated with this operator if they have non-trivial destructors.

Parameters
:   |  |  |
    | --- | --- |
    | nbytes | Number of bytes to allocate. |

Returns
:   Pointer to allocated bytes.

Definition at line 63 of file chainable.hpp.

|  |  |  |  |  |
| --- | --- | --- | --- | --- |
| virtual void stan::agrad::chainable::set\_zero\_adjoint | ( |  | ) |  |

inlinevirtual

Set the value of the adjoint for this chainable to its initial value.

Reimplemented in stan::agrad::vari.

Definition at line 51 of file chainable.hpp.

---

The documentation for this class was generated from the following file:

- src/stan/agrad/rev/chainable.hpp


---

[ Stan Home Page ]

*© 2011–2014,
Stan Development Team.*
